# A review of the genus *Sinocentrus* Yuan (Hemiptera, Membracidae, Centrotinae) with description of a new species from China

**DOI:** 10.3897/zookeys.886.36672

**Published:** 2019-11-05

**Authors:** Feng-E Li, Lin Yang, Jian-Kun Long, Zhi-Min Chang, Xiang-Sheng Chen

**Affiliations:** 1 Institute of Entomology, Guizhou University, Guiyang, Guizhou, 550025, China Guizhou University Guiyang China; 2 The Provincial Special Key Laboratory for Development and Utilization of Insect Resources of Guizhou, Guizhou University, Guiyang, Guizhou, 550025, China Guizhou University Guiyang China; 3 College of Animal Science, Guizhou University, Guiyang, Guizhou, 550025, China Guizhou University Guiyang China

**Keywords:** distribution, morphology, taxonomy, treehopper

## Abstract

A new species of the treehopper genus *Sinocentrus* Yuan, *S. brevicornis* Li & Chen, **sp. nov.** from China, is described and illustrated. A checklist and key to species of the *Sinocentrus* are provided.

## Introduction

The treehopper genus *Sinocentrus* was established by Yuan (Yuan and Chou 2002) with only its type species, *S. sinensis* Yuan, 2002, known from one female specimen. The genus was originally placed in the tribe Leptocentrini by Yuan and Chou (2002), but Wallace and Deitz (2004) moved *Sinocentrus* to *incertae sedis*, because specimens were not examined in their study and the morphological characteristics were confounding.

Herein, a new species, *Sinocentrus brevicornis* Li & Chen, sp. nov. from China, is described and illustrated. As a result of this act, the genus *Sinocentrus* now contains two species. A key based on morphological characteristics to distinguish species is provided as well as a map of their geographic distributions.

## Materials and methods

General morphological terminology follows Deitz (1975) and Dietrich et al. (2001) except morphology of the female genitalia, which follows Mejdalani (1998). Dry male specimens were used for the descriptions and illustrations. External morphology was observed under a stereoscopic microscope and characters were measured with an ocular micrometer. Measurements are given in millimeters; body length was measured from the apex of the head to the apex of the forewing in repose. Habitus photographs were taken using a NIKON SMZ 25 digital camera and multiple layers were stacked using Helicon Focus 6. The genital segments of the specimens examined were macerated in 10% NaOH and drawn from preparations in glycerin jelly using a Leica MZ 12.5 stereomicroscope. The photographs and the illustrations were imported into Adobe Photoshop CS5 for plate composition and labeling.

The type specimens examined are deposited in the Institute of Entomology, Guizhou University, Guiyang, Guizhou Province, China.

## Taxonomy

### Sinocentrus

Taxon classificationAnimaliaHemipteraMembracidae

Yuan, 2002

DF297E78-FFD7-5D4A-9519-57A1FEB956F7

Sinocentrus Yuan, 2002: 170.

#### Type species.

*Sinocentrus sinensis* Yuan, 2002: 170, by redescription.

#### Diagnosis.

Large sized. Frontoclypeus distinct. Suprahumeral horns narrow, acuminate, horizontally extended laterally, width between their apex ca 0.5 to 1.0 times body length. Pronotum highly developed, with anterior part strongly inflated and evenly rounded in profile, metopidium vertical, glabrous and minutely punctate without obvious pubescence. Posterior pronotal process strongly elevated above scutellum at base, slender, elongate, evenly tapered toward apex, straight or slightly sinuate, lateral and dorsal carina well developed, apex extended beyond forewing clavus. Scutellum entirely exposed, posterior margin emarginate. Basal one-fifth of forewing with opaque sclerotization, veins M and Cu fused basally to approximately one-fifth to one-fourth of wing length then strongly divergent, veins M+Cu and R fused basally, with 1 m-cu, 2 r-m, and 1 s crossveins. Hindwing vein R branched into R_1_, R_2+3,_ and R_4+5_, vein M branched into M_1+2_ and M_3+4_, R_4+5_ and M_1+2_ veins not fused (four apical cells present), 1 r-m and 1 m-cu crossveins present, apical limbus broad, with wrinkles. Metathoracic trochanter without spines, tibia with 3 rows of cucullate setae.

#### Remarks.

This genus can be distinguished from other oriental Centrotinae genera by the following characters: pronotum highly developed, strongly inflated with anterior part evenly rounded, glabrous with minute punctures and no obvious pubescence, suprahumeral horns extended laterad, posterior pronotal process elevated far above scutellum, scutellum emarginate.

#### Distribution.

China (Hainan, Yunnan) (Fig. 29).

### Checklist and distributions of species of *Sinocentrus* Yuan, 2002

*S. brevicornis* Li & Chen, sp. nov.; China (Hainan)

*S. sinensis* Yuan, 2002; China (Yunnan); elevation: 1600 m.

### Key to species of the genus *Sinocentrus* Yuan

**Table:** 

1	Forewing veins yellow to light brown, apical limbus hyaline; suprahumeral horns long, width between suprahumeral horns apices nearly as long as body length; posterior pronotal process curved near midlength; scutellum short, width greater than length (Figs 24–28)	***S. sinensis* Yuan**
–	Forewing veins black, contrasting with pale membrane, apical limbus black; Width suprahumeral horns short, width between suprahumeral horns apices nearly half length of body; posterior pronotal process nearly straight throughout length; scutellum long, longer than wide (Figs 1–6)	***S. brevicornis* sp. nov.**

### Sinocentrus brevicornis

Taxon classificationAnimaliaHemipteraMembracidae

Li & Chen sp. nov.

0F416316-4D26-546E-8578-BDD13EA0550D

http://zoobank.org/73BE02FB-C98E-4D45-AC88-D597817F3597

Figs 1–6 , 7–17 , 18–23

#### Type material.

***Holotype***: ♂, CHINA: Hainan, Bawangling, 29 April 2017, Hong-Xing Li. ***Paratypes***: 2♀♀, same data as holotype.

#### Description.

Body length: male 8.1 mm (*N* = 1), female 8.9–9.3 mm (*N* = 2); forewing length: male 7.3 mm (*N* = 1), female 7.3–7.9 mm (*N* = 2); width between humeral angles apices: male 3.3 mm (*N* = 1), female 3.5–3.8 mm (*N* = 2); width between suprahumeral horns apices: male 4.6 mm (*N* = 1), female 4.6–5.2 mm (*N* = 2).

***Coloration* .**

General color black with scattered yellow setae. Eyes pale brown with yellow border in males, pale yellow with black markings in females (Figs 5, 6), ocelli yellow hyaline. Basal one-third of scutellum dark brown, covered with yellowish-white setae, preapical region yellowish-brown with apical white. Forewing pale yellow hyaline, one-fifth of basal, veins and apical limbus black. Hindwing veins pale brown. Thorax black with pale yellowish pubescence; coxae black with yellow pubescence; trochanter, femur, tibia and tarsus yellowish-brown and tarsal claw dark brown. Abdomen reddish-brown with yellow pubescence, basal part of abdomen with a yellow spot of pubescence, apices of terga and sterna pale yellowish.

**Figures 1–6.**
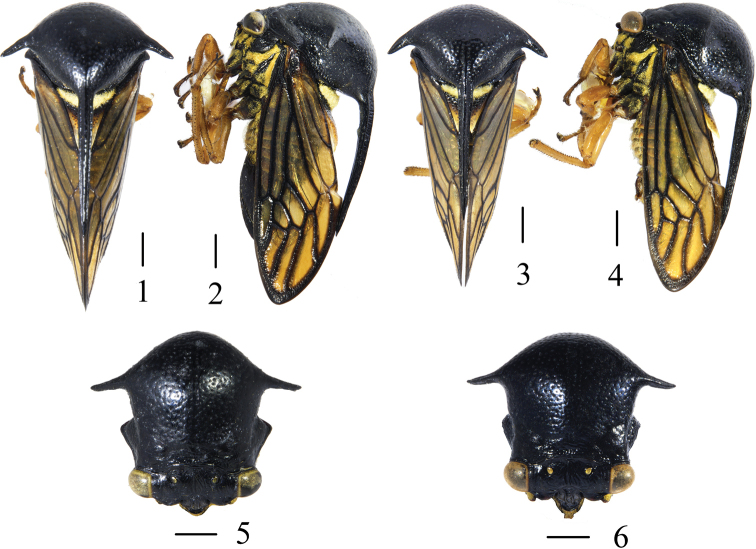
*Sinocentrus brevicornis* Li & Chen, sp. nov. **1** female habitus, dorsal view **2** same, lateral view **3** male habitus, dorsal view **4** same, lateral view **5** head and pronotum anterior view, female **6** head and pronotum anterior view, male. Scale bars: 1 mm.

***Head and thorax*.** Head in anterior view wider than long, ratio: 2.11:1. Vertex with dorsal and ventral margins slightly arcuated and wave-shaped respectively, with wrinkles on surface and a weak median longitudinal carina. Eyes and ocelli oval, ocelli slightly closer to inner margins of eyes less than to each other. Frontoclypeus distinct and trilobed, margin with sparse setae, apices of lateral and median lobes on same plane, more than half of median lobe extending beyond towards ventral margin of vertex, and apex dorsally slightly upturned. Apex of metopidium convex in anterior view. Posterior pronotal process ending at more than half of forewing cell M_3+4_ (last apical cell). Humeral angles triangular with apices somewhat blunt. Suprahumeral horns short, width between horns apices nearly half length of body. Scutellum humped basally, large punctures present, longer than wide, apex extended antero-dorsally male, curved ventrally in female (Figs 2, 4), posterior margin deeply emarginate. Mesothoracic femur without ablateral and adlateral cucullate setae. Metathoracic leg cucullate setae row II irregular.

**Figures 7–17.**
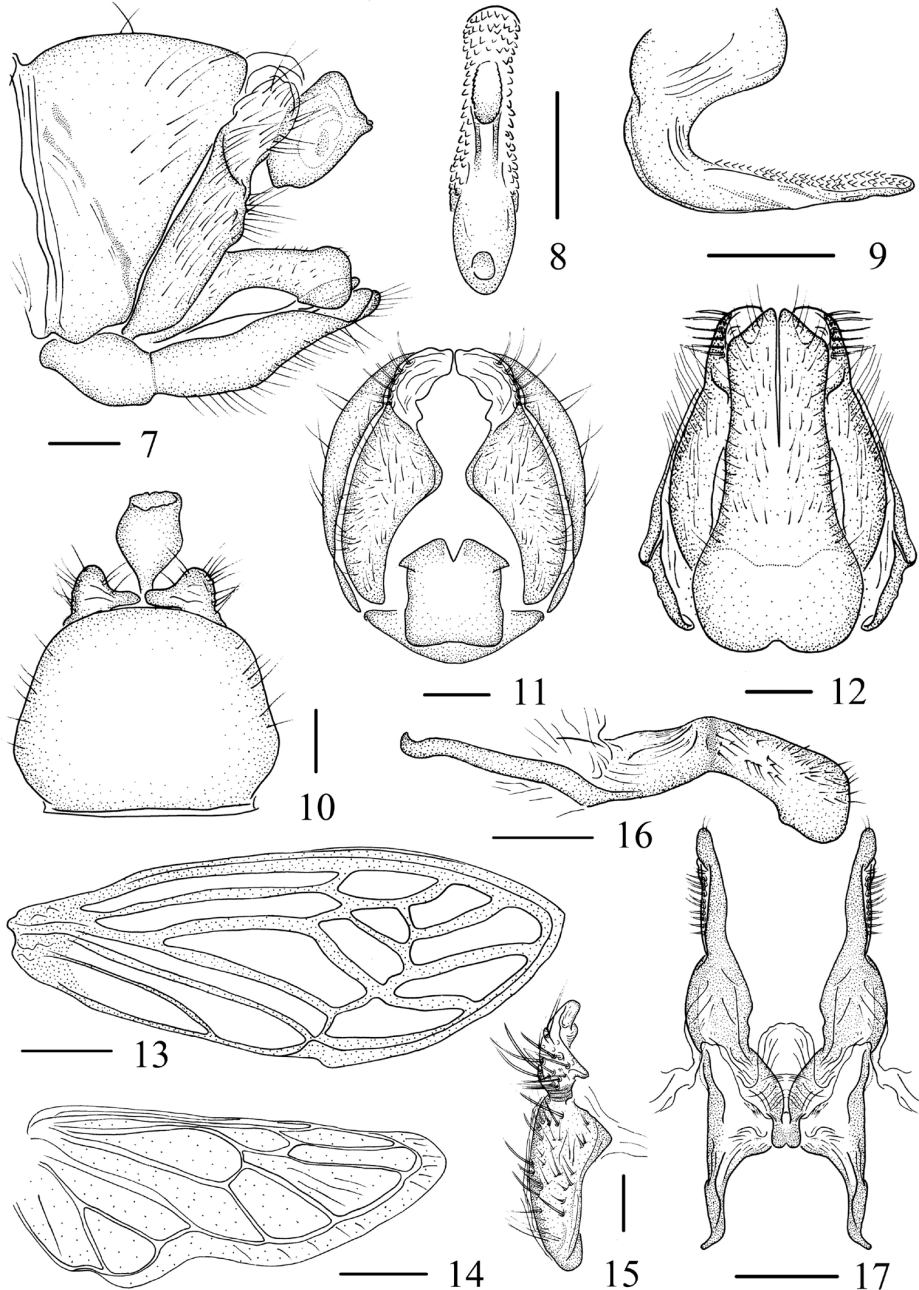
*Sinocentrus brevicornis* Li & Chen, sp. nov. **7** male genitalia, lateral view **8** aedeagus, posterior view **9** aedeagus, lateral view **10** male genitalia, dorsal view **11** male genitalia, posterior view **12** male genitalia, ventral view **13** forewing **14** hindwing **15** lateral plate **16** style, right lateral view **17** style, dorsal view. Scale bars: 0.2 mm (**7–12, 15–17**), 1 mm (**13–14**).

***Male genitalia*.** Pygofer (Figs 7, 11) nearly trapezoidal in lateral view; sternite IX (Fig. 12) depressed medially in ventral view. Anal tube cylindrical-shaped in lateral view. Lateral plate (Figs 7, 11, 15) with membranous dorsoapical lobe extending dorsally, slightly arcuate with setae; part of other surface with setae, margin incurved in posterior view. Basal half of subgenital plates fused, apex acute, obliquely truncate, distributed evenly setae in ventral view (Fig. 12). Style (Figs 16, 17) clasp oriented laterally, one-third compressed apically, weakly angled ventrally, lateral surface with setae; style shank with arch at central section. Base of connective W-shaped, distal part membranous and weakly depressed. Aedeagus in lateral view (Fig. 9) nearly C-shaped, apical four-fifths of surface and margin with reverse serrations, the opening at middle-upper of aedeagus, ovoid.

**Figures 18–23.**
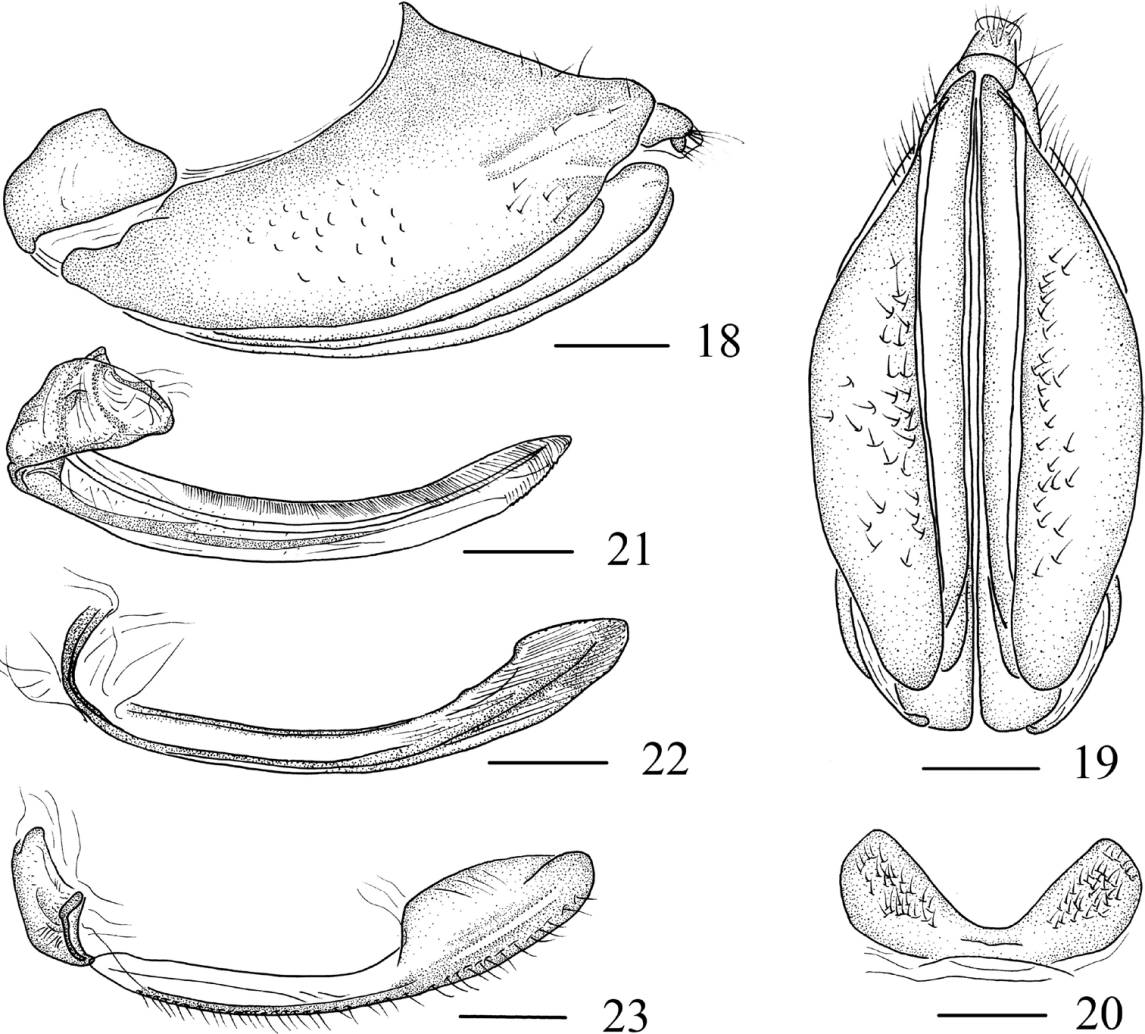
*Sinocentrus brevicornis* Li & Chen, sp. nov. **18** female genitalia, lateral view **19** female genitalia, ventral view **20** sternite VII, ventral view **21** valvifer I and valvulae I, lateral view **22** valvulae II, lateral view **23** valvifer II and gonoplac, lateral view. Scale bar: 0.5 mm.

***Female genitalia*.** Sternite VII (Fig. 20) in ventral view with posterior margin concave, lateral margins convex and surface with setae. Pygofer (Figs 18, 19) in lateral view irregularly quadrilateral, with setae; in ventral view oblong, base slightly acute. Anal tube (Figs 18, 19) small and oval. Valvifer I (Fig. 21) semicircular and thin; valvulae I knife-shaped, apical three-fourths of dorsal surface sculptured, ventral surface of the apex with a row of small toothed processes. Valvifer II (Fig. 23) shoe-shaped in lateral view, dorsal margin membranous. Basal part of valvulae II (Fig. 22) connected to the apex of “sole”, ramus slender, parallel-sided and evenly curved in basal two-thirds, apex slightly broadened with two indistinct and widely spaced dorsal preapical tooth processes; the one-third of apical broaden slightly. Gonoplac (Fig. 23) connected to base of “sole”, apical third expanded, ventral margin with setae.

#### Distribution.

China (Hainan).

#### Etymology.

The word “*brevicornis*” is derived from the Latins words “*brevi*-” and “*cornu*”, referring to having the short suprahumeral horns.

#### Remarks.

This species is similar to *S. sinensis* Yuan, 2002, but differs from the latter in: (1) forewing veins black and apical limbus black (veins yellow to light brown, apical limbus hyaline in *S. sinensis*) (2) suprahumeral horns short, width between suprahumeral horns apices shorter than body length (as long as body length in *S. sinensis*); (3) posterior pronotal process nearly straightly (concave medially in *S. sinensis*); (4) scutellum longer than wide (wider than long in *S. sinensis*); (5) apex of posterior pronotal process not reaching M_3+4_ veins (exceeding M_3+4_ veins in *S. sinensis*).

### Sinocentrus sinensis

Taxon classificationAnimaliaHemipteraMembracidae

Yuan, 2002

074481DF-471C-5038-857A-48AFE38F7BD3

Figs 24–28

Sinocentrus sinensis Yuan, 2002: 170, by original designation.

#### Description.

***Coloration*.** General color reddish-brown with golden setae (Fig. 28). Head blackish-brown. Ocelli pale yellow. Eyes yellow-brown. Forewing dark brown; A_1_, A_2_, A_3,_ and Cu_1_ white hyaline; Sc, R, and M brown; Cu and A pale yellow. Hindwing with veins pale yellow. Thorax dark brown. Legs reddish-brown except with tarsi yellow. Abdomen dark brown.

**Figures 24–28.**
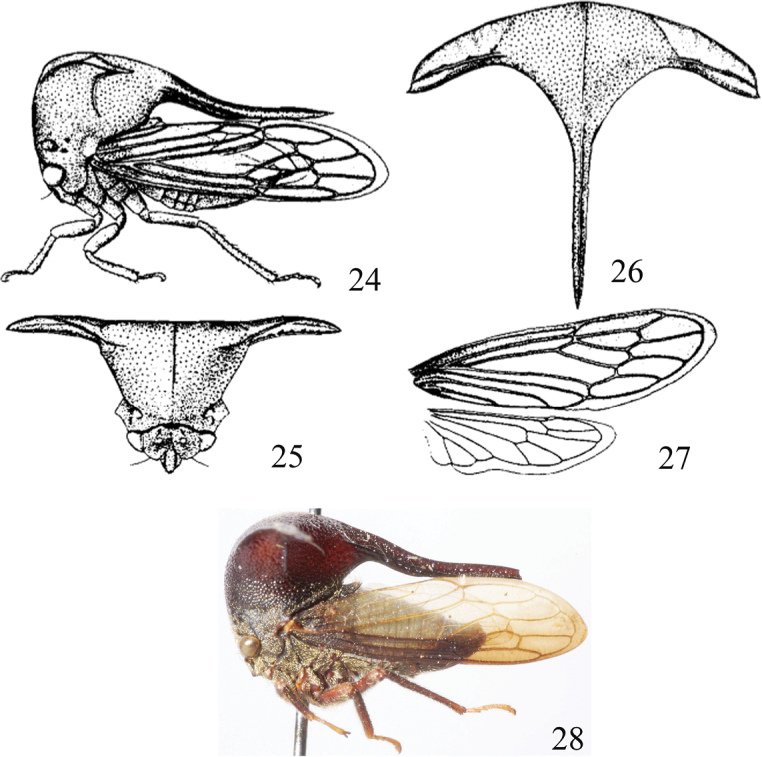
*Sinocentrus sinensis* Yuan, 2002 **24** female habitus, lateral view **25** head and pronotum, anterior view **26** female habitus, dorsal view **27** forewing and hindwing **28** holotype, female, habitus, lateral view, photo by Robert L. Snyder from the treehopper website http://treehoppers.insectmuseum.org Note: **24–27** from Fauna Sinica. Insecta Vol. 28, 171 pp, figure 66.

***Head and thorax*.** Head wider than long. Vertex with dorsal margin arched and ventral margin oblique. Eyes oval. Ocelli hyaline, slightly closer to the inner margin of eyes less than to each other. Frontoclypeus distinct and trilobed, the apex of lateral lobes and the median lobes on the same plane, two-thirds of median lobe extending beyond towards ventral margin of vertex. Pronotum with dense setae and punctures. Humeral angles large, apices blunt. Suprahumeral horns leaflike pyramidal, horizontally extended laterally, width between suprahumeral horns apices nearly as long as body length (Figs 24–25). Nearly median part of the posterior pronotal process concave and touching forewing, apical upward, with four carinas. Scutellum short, wider greater than length, posterior margin round emarginate. Forewing with opaque sclerotization at basal one-eighth, Venation similar to that of *S. brevicornis*. Metathoracic trochanter without spines and tibia with 3 rows of cucullate setae.

**Male.** Unknown.

#### Distribution.

China (Yunnan).

#### Note.

While holotype was not examined, an online image of the holotype (Fig. 28) and detailed Chinese description were available.

**Figure 29.**
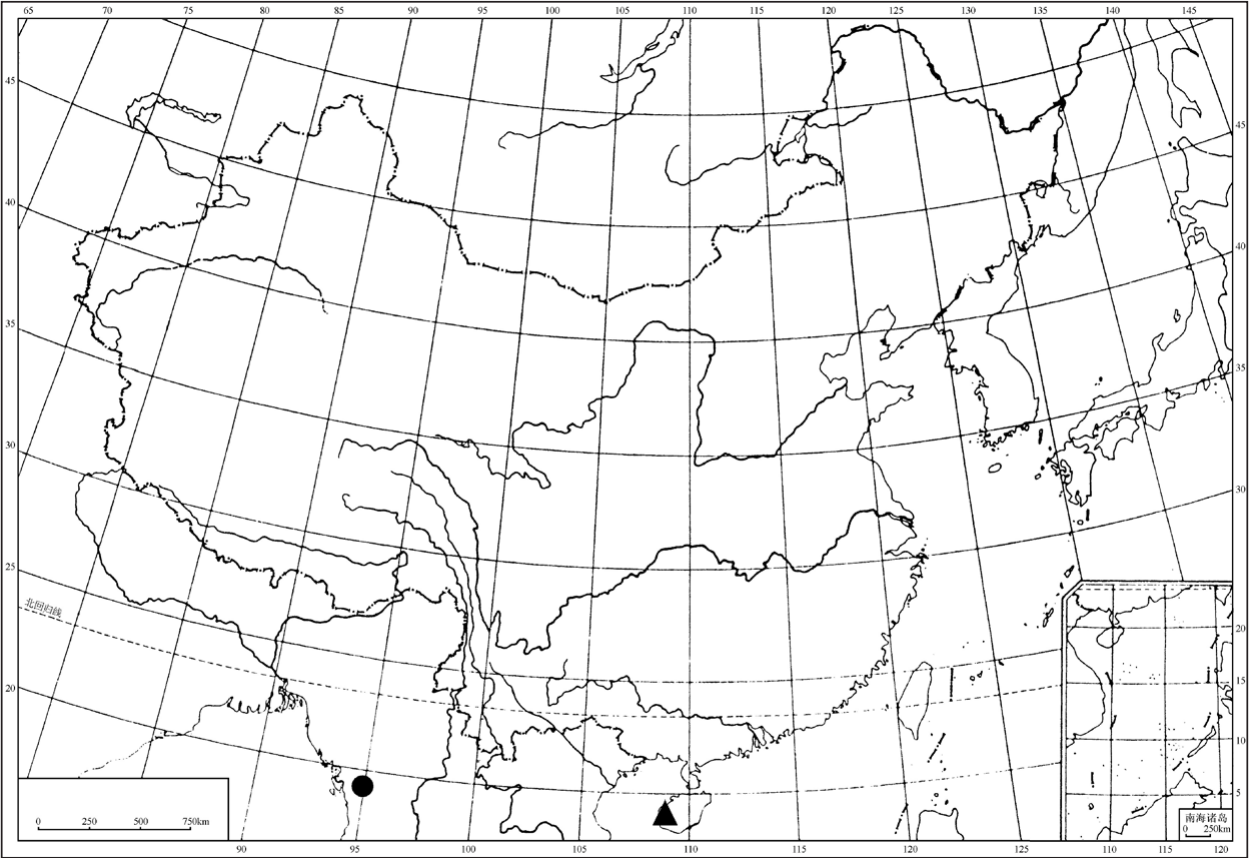
Geographic distributions of *Sinocentrus* species: *S. sinensis* (circle); *S. brevicornis* Li & Chen, sp. nov. (triangle).

## Discussion

In their phylogeny and genus-level revision of Centrotinae, Wallace and Deitz (2004) were unable to examine specimens of *Sinocentrus* and treated the genus as Centrotinae, *incertae sedis*.

We provide the following additional details on *Sinocentrus*: (1) frontoclypeus distinct (indistinct in Centrotypini); (2) posterior pronotal process elevated far above the scutellum, entirely exposed (straight at base, partially covers the scutellum in Centrotypini); (3) male lateral plate with short dorsoapical lobe extending dorsally, style clasp angled ventrally; style shank with arch at central section (angled dorsally; style shank with significant arch medially in Centrotypini); (4) mesothoracic femur without ablateral and adlateral cucullate setae; metathoracic leg cucullate setae row II irregular. Although the above characteristics can suggest that the genus is related to Leptocentrini, the shape of the female second valvulae closely align *S. brevicornis* with the tribe Centrotypini. Given these mixed affinities, we follow Wallace and Deitz, in treating *Sinocentrus* as Centrotinae, *incertae sedis*. Proper tribal placement may be affirmed by future phylogenetic analyses of combined morphological and molecular data.

## Supplementary Material

XML Treatment for Sinocentrus

XML Treatment for Sinocentrus brevicornis

XML Treatment for Sinocentrus sinensis

## References

[B1] Deitz LL (1975) Classification of the higher categories of the New World treehopper (Homoptera: Membracidae). North Carolina Agricultural Experiment Station Technical Bulletin 225: 1–177.

[B2] Deitz LL Wallace MS Rothschild MJ (2010) [and updates] Treehoppers: Aetalionidae, Melizoderidae, and Membracidae (Hemiptera). http://treehoppers.insectmuseum.org [accessed on 13 April 2019]

[B3] Dietrich CH McKamey SH Deitz LL (2001) Morphology-based phylogeny of the treehopper family Membracidae (Hemiptera: Cicadomorpha: Membracoidea). Systematic Entomology 26: 213–239. 10.1046/j.1365-3113.2001.00140.x

[B4] Mejdalani G (1998) Morfologia externa dos Cicadellinae (Homoptera, Cicadellidae): comparação entre *Versigonalia ruficauda* (Walker) (Cicadellini) e Tretogonia cribrata Melichar (Proconiini), com notas sobre outras espécies e análise da terminologia. Revista Brasileira de Zoologia 15: 451–544. 10.1590/S0101-81751998000200015

[B5] Wallace MS Deitz LL (2004) Phylogeny and systematics of the treehopper subfamily Centrotinae (Hemiptera: Membracidae). Memoirs on Entomology International 238.

[B6] Yuan F Chou Y (2002) Fauna Sinica. Insecta (Vol. 28). HomopteraMembracoidea. Science Press, Beijing, 590 pp.

